# Inevitable fates? The recovery potential of small populations

**DOI:** 10.1098/rspb.2026.2436

**Published:** 2026-11-04

**Authors:** Donal Smith, John G. Ewen, Rohan H. Clarke, Zarah Ann Wessels, Samuel T. Turvey

**Affiliations:** ^1^ School of Biological Sciences, Monash University - Clayton Campus, Melbourne, Victoria, Australia; ^2^ Institute of Zoology of the Zoological Society of London, London, UK

**Keywords:** extinction, minimum viable population, population recovery, rarity, small population, triage, conservation

## Abstract

The recovery prospects of species reduced to critically small numbers are frequently seen in a pessimistic light. Even if conservation efforts could successfully tackle drivers of severe decline, small population effects would be expected to inhibit population growth. Accounts of dramatic conservation recoveries seem to challenge this narrative, but without a broader examination encompassing all actions and outcomes, the representativeness of success stories alone is unclear. Here, we conduct an observational study evaluating all 147 bird and mammal species that have been below 50 individuals in the wild at any point since 1950, of which 141 had sufficient elapsed time to assess an outcome. We characterized the history, if any, of conservation action in these species, and used Bayesian multi-level logistic regression to model its association with subsequent population status. Most species subject to direct conservation recovered to at least 100 individuals, with the highest modelled recovery probabilities associated with those subject to a combination of actions to limit external drivers of decline and directly manipulate populations. These findings indicate a striking potential for recovery even in critically small populations and serve as an empirically grounded counterpoint to advocates of abandonment of such species under a conservation triage framework.

‘Some of the birds are among the rarest in the world, and the Mauritius kestrel *Falco punctatus* may well be the rarest. This small forest-dwelling falcon, poised on the brink of extinction, has a known population of only six… the chances for its survival in the wild seem to be virtually nil. Two other forest dwellers, the pink pigeon *Columba mayeri*, one of the island's most beautiful birds, and the parakeet *Psittacula echo*, seem doomed’ [1].

‘We might abandon the Mauritius kestrel to its all-but inevitable fate, and use the funds to proffer stronger support for any of the hundreds of threatened bird species that are more likely to survive’ [2].

## 1. Introduction

The bleak assessments offered by Stanley Temple and Norman Myers reflect the perilous state faced by Mauritian forest birds in the 1970s. A suite of anthropogenic pressures, including introduced mammalian predators, habitat degradation and pesticide use, had driven the Mauritius kestrel (*Falco punctatus*), pink pigeon (now *Nesoenas mayeri*) and echo parakeet (now *Alexandrinus eques*) to the brink of extinction [3]. With fewer than 50 individuals of each species remaining, the prospects for recovery or even persistence appeared low: conservation of these species would require not only addressing the external factors that had driven the populations to such small numbers, but also confronting the effects of smallness itself.

Once populations become critically small, they are expected to become more vulnerable to demographic stochasticity, environmental fluctuation, Allee effects, inbreeding depression and loss of genetic diversity through drift [4,5]. Each of these effects further reduces population size and, becoming ever more acute as size decreases, acts synergistically in a feedback loop pulling a population into an ‘extinction vortex’ towards zero [6]. Genetic and demographic concerns underpin generalized thresholds below which populations may be considered non-viable: effective population sizes (*N*_e_) of 50 and 500 for short-term and long-term genetic viability respectively (the ‘50/500 rule’ [7,8]), since revised upward to 100 and 1000 [9], with census sizes of at least 5000 adults proposed as necessary to avoid a turning point towards extinction [4].

What does this mean for remnant populations of threatened species far below these thresholds? Severely bottlenecked populations are often perceived as fundamentally compromised in their fitness and capacity to respond to environmental change, showing, for example, greater levels of reproductive failure [10,11] and vulnerability to novel pathogens [12]. Consequently, their ostensibly poor recovery prospects can make them candidates for inaction under a conservation triage framework, in which finite resources are directed away from species where success seems unlikely or disproportionately costly. Conservation triage is contentious [13–15], with varying biological, economic or logistical definitions put forward to determine when a project should be avoided or abandoned [4,16]. Proponents may regard small populations as inherently unsavable [4], or as recoverable in principle but unreasonable to fund [17,18]; often they are proposed as candidates for inaction without explicit statement of whether recovery is possible [15,19,20].

Although it is sometimes acknowledged that small populations that have since recovered would have been lost if a hard-line triage approach had been applied [21], triage thinking has hampered conservation efforts that eventually succeeded [22], and it is considered a contributor to the scaling back of active conservation efforts prior to the extinction of the baiji (or Yangtze River dolphin, *Lipotes vexillifer*) [18,23]. The concept that some small populations cannot be recovered, and should therefore be abandoned to their fate, continues to influence conservation planning for many highly threatened species [15,20,24].

However, how do we know when, or if, a cause is lost? Almost half a century after Myers’ and Temple's pessimistic prognoses [1,2], the pink pigeon, echo parakeet and Mauritius kestrel are now reclaiming the Mauritian forests, each with populations in the hundreds [25–27]. These remarkable recoveries are attributed to conservation interventions targeted towards mitigating the external factors limiting population growth—including control of invasive mammalian predators, provision of supplementary food and artificial nest sites—combined with interventions to directly manipulate populations, including cross-fostering eggs, *ex situ* care and conservation translocations [25–27]. The Mauritius kestrel remains a challenging case, having declined again from 350–500 in the late 1990s to under 250 in 2021 [26], but the astonishing rebound from only four wild birds in 1974 is far from the ‘inevitable fate’ envisaged by past advocates for abandonment through triage.

These recoveries form part of a broader set of case studies about which much has been written. The California condor (*Gymnogyps californianus*) and European Bison (*Bison bonasus*), and the now-extinct baiji and po'ouli (*Melamprosops phaeosoma*), have become high-profile and sometimes controversial conservation icons [23,28–31]. Their conservation histories, however, have largely been examined case-by-case as cautionary or good-news stories. Several reviews have investigated correlates of success or failure across recovery programmes [32–36], but none has specifically targeted the recovery potential of extremely small populations, which may differ in their ability to respond to intervention. A diverse range of actions have been attempted to recover such species, from less direct actions such as legal protection, to direct actions mitigating limiting factors and manipulating populations using both *in situ* and *ex situ* approaches [15,35,37]. A clearer understanding of how these varying types of intervention, acting alone or in combination, mediate the inevitability of extinction as well as the possibility of recovery, is needed. Two major reviews of birds and mammals have identified species for which conservation may have been instrumental in avoiding extinction [38,39], but without a broader examination encompassing all outcomes from loss to recovery—where conservationists have tried and succeeded, tried and failed, or failed to try—the representativeness of success stories alone is unclear.

Here, we focus on all bird and mammal species whose populations have been below 50 individuals in the wild at any point since 1950 and examine the association between their history of conservation, if any, and their current status. Inevitably, our study is observational: conservation action was not allocated at random, and we therefore estimate associations between recorded action and subsequent population status rather than treatment effects. By conducting this analysis across all contemporary bird and mammal species that have found themselves so close to extinction, we contribute a comprehensive empirical assessment of the prospects of extinction and recovery in small populations.

## 2. Methods

### (a) Species inclusion criteria

We examined the association between conservation action and subsequent population status for bird and mammal species that have been extant with a wild census population below 50 individuals at any point since 1950, including species that subsequently exceeded this threshold and those that became extinct. Species were identified using the IUCN Red List of Threatened Species version 2025-2 [40] (hereafter the ‘Red List’), supplemented with key literature references as detailed below.

An *N*_e_ of 50 has frequently been cited in the conservation literature as the minimum threshold necessary to avoid extinction owing to the consequences of inbreeding [7,8], and the Red List converged on 50 ‘mature individuals’ marking a species as critically endangered under criterion D (hereafter ‘CR D’) owing to the deleterious effects of demographic stochasticity [41]. ‘Mature individuals’ *sensu* IUCN refers to those capable of reproduction [42]; this is neither census size nor *N*_e_, but accounts for the difference between total population and the component contributing to its perpetuation and growth. Owing to the impracticality of estimating either quantity for wild populations across a set of historical contexts, we used a census population of 50 as a marker of extreme demographic and genetic peril within these thresholds. Throughout this study, population size corresponds directly to census population size as reported in the relevant documentation. We followed Akçakaya *et al.* [43] and the IUCN Green Status [44] in using 1950 as a fixed starting point representing a proxy for the birth of modern conservation, after which most of the documented conservation history for most species is captured. Adopting these cut-offs was necessary to produce a manageable and transparent scope, but inevitably means that potentially important variation in undocumented population history remains uncharacterized by our study.

Extant species currently below 50 were identified by reviewing those assessed as CR D and filtering out those with census population sizes above 50. CR D species with no confirmed records since 1950 were also excluded. For species that have since improved and are no longer CR D, we compiled genuine downlists from CR D in Red List status change summaries between 2007 and 2025 [40] and between comprehensive assessments of mammals (1996, 2008) and birds (1988, 2020) [45]. For improvements not captured by the Red List process, we consulted key references on taxa that have come close to extinction [39,40] and those recovered from extinct in the wild status [45], plus cases encountered incidentally. Species currently assessed as extinct in the wild were treated as within scope. We also included all species categorized on the Red List as extinct whose last confirmed record was 1950 or later. To avoid including species that met the criteria too recently to assess an outcome, we excluded those that dropped below 50 within the longer of three generations or 10 years of their most recent assessment or population estimate, following the generation-calibrated time window used throughout the Red List [40]. Taxa were assessed at species level only, with taxonomy following Red List 2025-2 and applied retrospectively. Where source literature used superseded names, records were reconciled to the currently accepted Red List name.

### (b) Population and conservation action data

Population and conservation histories were compiled from Red List assessments in the first instance, supplemented where necessary by targeted searches of the academic and grey literature. To accommodate variation in precision, we binned current and historical population estimates into broad classes which served as outcome thresholds. The lowest recovery threshold was 100 individuals—a minimum doubling of the inclusion threshold—with remaining bands taken from CR D of the Red List, giving five classes: extinct, 1–99, 100–249, 250–999 and ≥1000. We recorded the estimated year of each species' initial drop below 50, year of extinction (using the year after the last confirmed record as a proxy), and years of entering each population class. Where timing could only be recorded as a range, we used the midpoint, testing sensitivity to this choice. Population data were included to 2025 where available.

### (c) Classification of conservation actions

The ‘actions in place’ component of Red List assessments is limited in scope and not mandatory [46], and timing is not part of systematic data reporting. We therefore assembled our dataset using the narrative text elements of Red List assessments, and follow-up targeted searches of the academic and grey literature. Because extent, duration and expected impact were not consistently reported, we recorded only the year of initiation of each action.

Actions were grouped into common types and then into three broad classes (figure 1): *limiting factor mitigation* attempts to address external factors driving decline or preventing growth, and includes predator control, nest box provision and habitat restoration where the restored elements are relevant to that species' growth. *Population manipulation* acts directly on the species to stimulate growth or prevent decline, and includes translocation, clutch manipulation and behavioural training. *Ex situ* care was also included here, but restricted to cases where initial capture was clearly for conservation purposes and/or where translocation back to the wild has occurred. Collection of animals for the pet trade was not counted unless and until a release was attempted. *Indirect action* comprises measures that are not expected to affect a population in and of themselves, but are largely instruments that seek to influence people. It includes laws, designations and public campaigns and is included only where specifically invoked in project documentation. These may create enabling conditions but typically require pairing with direct action, which is logged separately; for example, designation of a national park was classified as indirect, whereas building a fence within it to alleviate a threat relevant to the species was classified as limiting factor mitigation.

**Figure 1.**
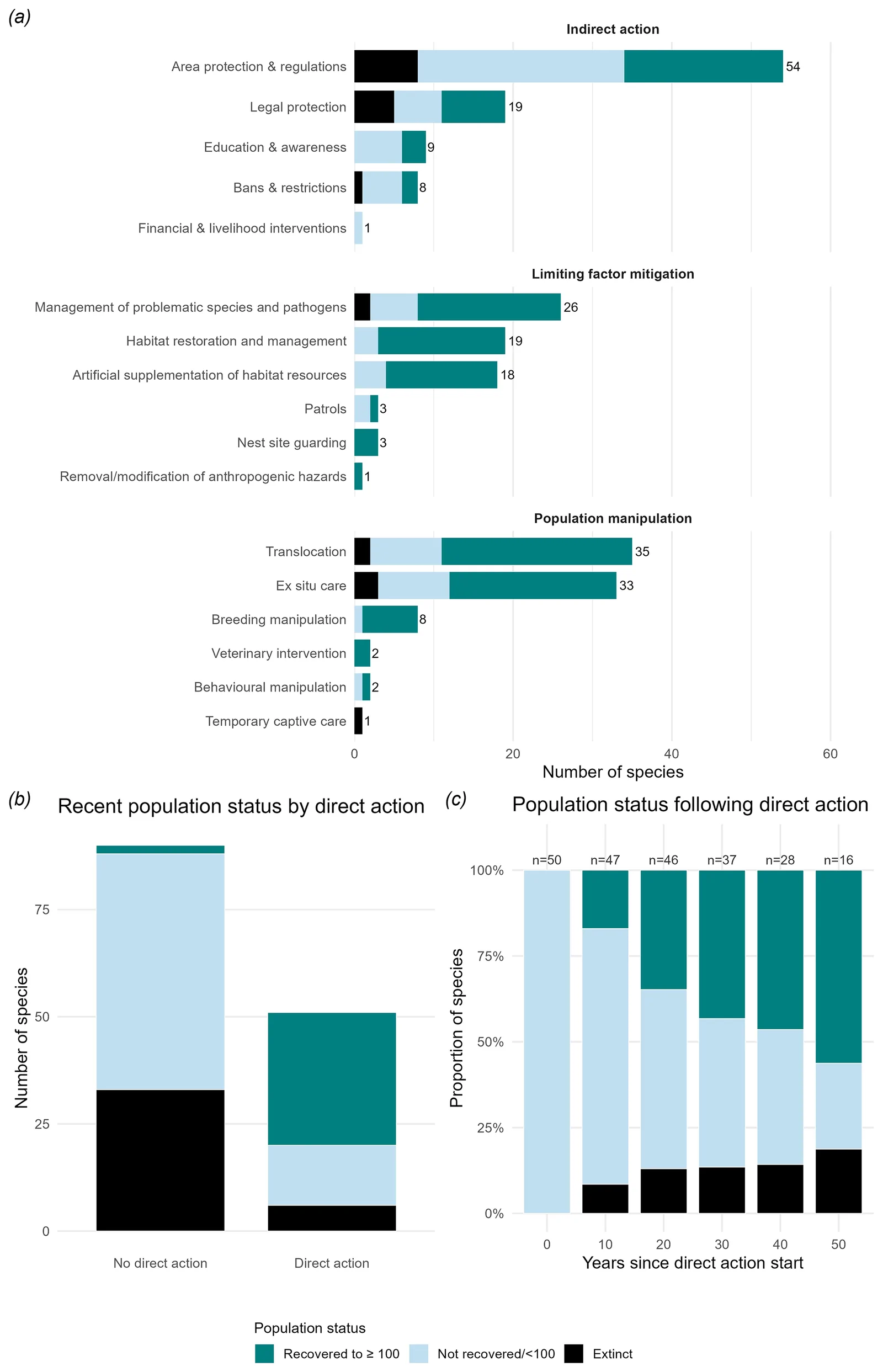
Population status of bird and mammal species with a wild population below 50 individuals at some point since 1950. (*a*) Conservation actions taken while populations were small or declining (i.e. before recovery above 100 or extinction, where applicable), coloured by most recent status; bar-end numbers give species counts. (*b*) Recent status of species that did and did not receive direct conservation action (limiting factor mitigation or population manipulation). (*c*) Population status by decade since direct conservation action began; each bar includes only species that have reached that interval, with status back-cast at each earlier decade (so all species in the 50-year bar also appear, at their earlier status, in every preceding bar).

Integrating information on limiting factors (e.g. threats) would be extremely valuable but raises quality and consistency concerns. Unlike conservation actions, which are specific real-world events, reported threats are ecological claims with varying evidential support. We were not confident that variation in reported threats reflects genuine variation in threats experienced. We therefore excluded threats from the primary analyses, but tested sensitivity to this decision (table 1).

**Table 1.** Specification of primary models and sensitivity analyses (*n* = 141 species, see main text for full sample definition). Unless stated otherwise, fixed-effect priors were normal (0, 1) on the log-odds scale. ‘CR D’ refers to IUCN Red List critically endangered status under criterion D (population size).

analysis	rationale	specification
primary models	test association between conservation action type and extinction/recovery, at multiple population-size thresholds	four Bayesian binary logistic models (extinction; recovery at ≥100, ≥250 and ≥1000 individuals). Fixed effects: three action categories, log-scaled generation length, taxonomic class; random intercept for taxonomic order. Actions initiated after a given threshold was reached were excluded from that model. Generation length: Red List assessment (birds); [47] (mammals, mean of extant congeners as proxy where extinct-taxon data missing)
prior sensitivity	assess sensitivity to prior choice	refit under ‘tight’ (normal (0, 0.5)) and ‘wide’ (normal (0, 2)) priors on the log-odds scale, versus default normal (0, 1)
source of inclusion outside Red List framework	address potential documentation bias from species included via non-Red-List literature	refit excluding species identified via non-Red-List sources (*n* = 13)
thinning of CR D species with no direct action	address concern that the group coded as below 50 and lacking any direct action may be inflated by precautionary Red List assessments (see §4)	refit with the no-direct-action CR D group reduced by random removal at 10%, 25% and 50%
possibly Extinct recoding	account for uncertain status of ‘Possibly Extinct’ species	refit recoding all Possibly Extinct species (*n* = 22) as Extinct (worst-case scenario). Only applied to extinction model
Red List threat category inclusion	test sensitivity to excluding threat data (omitted from primary models owing to unreliable evidential basis and potential documentation bias)	refit with added covariate: count of distinct ongoing or historical threats causing ‘slow, significant declines’ or worse over the majority of the population listed in Red List assessments
action and population timings variation	some action and population dates could only be recorded as a range; primary models use the range midpoint.	refit under two extremes that maximize the chance of an action reclassification: action dates to their earliest bound and population threshold-crossing dates to their latest bound, and the reverse.

### (d) Statistical analyses

We examined whether the three broad classes of conservation action (limiting factor mitigation, population manipulation and indirect action) were associated with extinction avoidance and population recovery to multiple thresholds. Four Bayesian binary logistic regression models were fitted (table 1), using the brms package [48] with the CmdStanR backend [49] and weakly informative priors (default normal (0,1). Analyses were conducted in R version 4.5.2 [50]; full code is available in the supplementary material. To visualize differences between action-type combinations, we generated population-average predicted probabilities (marginal over taxonomic order, at mean log generation length) for each of the eight combinations, separately for birds and mammals. Because the year a population dropped below 50 was unrecorded for most unrecovered species lacking action, a time-to-event analysis was considered inappropriate owing to the lack of a defensible starting point across all species.

## 3. Results

### (a) Species and conservation actions

We found that 147 bird and mammal species (98 birds and 49 mammals) have been extant with a wild population size below 50 at some point after 1950. A further 10 species were screened out owing to errors in Red List assessments (three) or assessments superseded by more recent publications (seven; supplementary material, table S1a). Six qualifying species were excluded because their drop below 50 occurred within the longer of three generations or 10 years of their most recent assessment or population estimate (supplementary material, table S1b), leaving 141 species (96 birds and 45 mammals) for analysis.

Of these, 80 species (57%) were subject to some form of conservation attention (figure 1), but only 51 (36%) to direct action (limiting factor mitigation or population manipulation). Area protection was the most common indirect action (51 species). The most common limiting factor mitigations were management of problematic species and pathogens (26 species), habitat restoration and management (19 species) and artificial supplementation of habitat resources such as supplementary food, nest boxes and canopy bridges (18 species). *Ex situ* care (33 species) and translocation (35 species) were the most common population manipulations.

### (b) Outcomes

Of the 141 species, 39 (28%) have subsequently gone extinct, 69 (49%) remain below 100 (with 21 of these regarded as possibly extinct and one as possibly extinct in the wild) and 33 (23%) have recovered to 100 or more (six in the 100–249 band, 16 in 250–999 and 11 at 1000 or more). The current Red List statuses of those that have recovered above 100 range from the most threatened to the least: with one species classified as extinct in the wild, seven as critically endangered, 11 as endangered, eight as vulnerable, three as near threatened and three as least concern. Population low points and recent population estimates of all recovered species are summarized in supplementary material, table S2. Eight species recovered from bottlenecks lower than 20 individuals, the Mauritius kestrel from just four. A further seven recovered from no wild individuals at all, persisting only in *ex situ* care at their nadir and now numbering from 340 (black-footed ferret, *Mustela nigripes*) to over 6000 (European bison).

Outcomes for species subject to direct action contrasted starkly with those receiving none or only indirect action (figure 1*b*). Of 51 species receiving direct action, 31 (61%) reached ≥100 individuals and six (12%) went extinct. Just two of the 37 species subject to limiting factor mitigation (5%) went extinct. By contrast, of 90 species receiving no direct action, two (2%) recovered above 100 and 33 (37%) went extinct. Among the 31 species that recovered following direct action, the median interval between action initiation and recovery was 18 years (range 1–57, interquartile range (IQR) 22). Populations above 100 became increasingly common with time since action initiation (figure 1*c*).

### (c) Modelled associations

Bayesian modelling of extinction and recovery aligned with the raw summaries, with the same pattern holding at every recovery threshold examined (table 2, figure 2). Direct actions showed strongly positive associations with recovery to ≥100 and ≥250. Associations were weaker at ≥1000, where the credible interval for population manipulation crossed one and that for limiting factor mitigation only marginally excluded it. Indirect action alone showed no association with recovery. Across both birds and mammals, limiting factor mitigation and population manipulation were associated with substantially higher posterior probabilities of recovery to ≥100 and ≥250, and were strongest in combination: predicted probability of recovery to ≥100 was 74% in birds and 71% in mammals with both action types, against 6% and 5% respectively with no action.

**Table 2.** Posterior odds ratios (ORs) from Bayesian regression models estimating associations between conservation action classes and species outcomes across an extinction/recovery spectrum. Values are posterior medians with 95% credible intervals. ORs below one indicate reduced odds of the outcome; ORs above one indicate increased odds. * Fragile under sensitivity testing; see text.

term	extinction (OR)	recovery ≥100 (OR)	recovery ≥250 (OR)	recovery ≥1000 (OR)
indirect action	0.5 (0.21–1.13)	1.16 (0.41–3.16)	1.22 (0.42–3.48)	1.78 (0.52–6.32)
limiting factor mitigation	0.32 (0.1–1.02)	8.71 (3.09–25.63)	7.56 (2.51–24.83)	3.81 (1.01–15.86)*
population manipulation	0.68 (0.25–1.85)	5.53 (2.09–14.84)	5.94 (2.03–18.41)	3.23 (0.87–12.92)
generation length (per 1 s.d. in log)	0.61 (0.35–1.02)	1.42 (0.82–2.47)	1.5 (0.82–2.77)	1.31 (0.63–2.67)
class (mammals versus birds)	2.14 (0.72–7.91)	0.85 (0.28–2.52)	0.69 (0.22–2.12)	0.86 (0.21–3.03)

**Figure 2.**
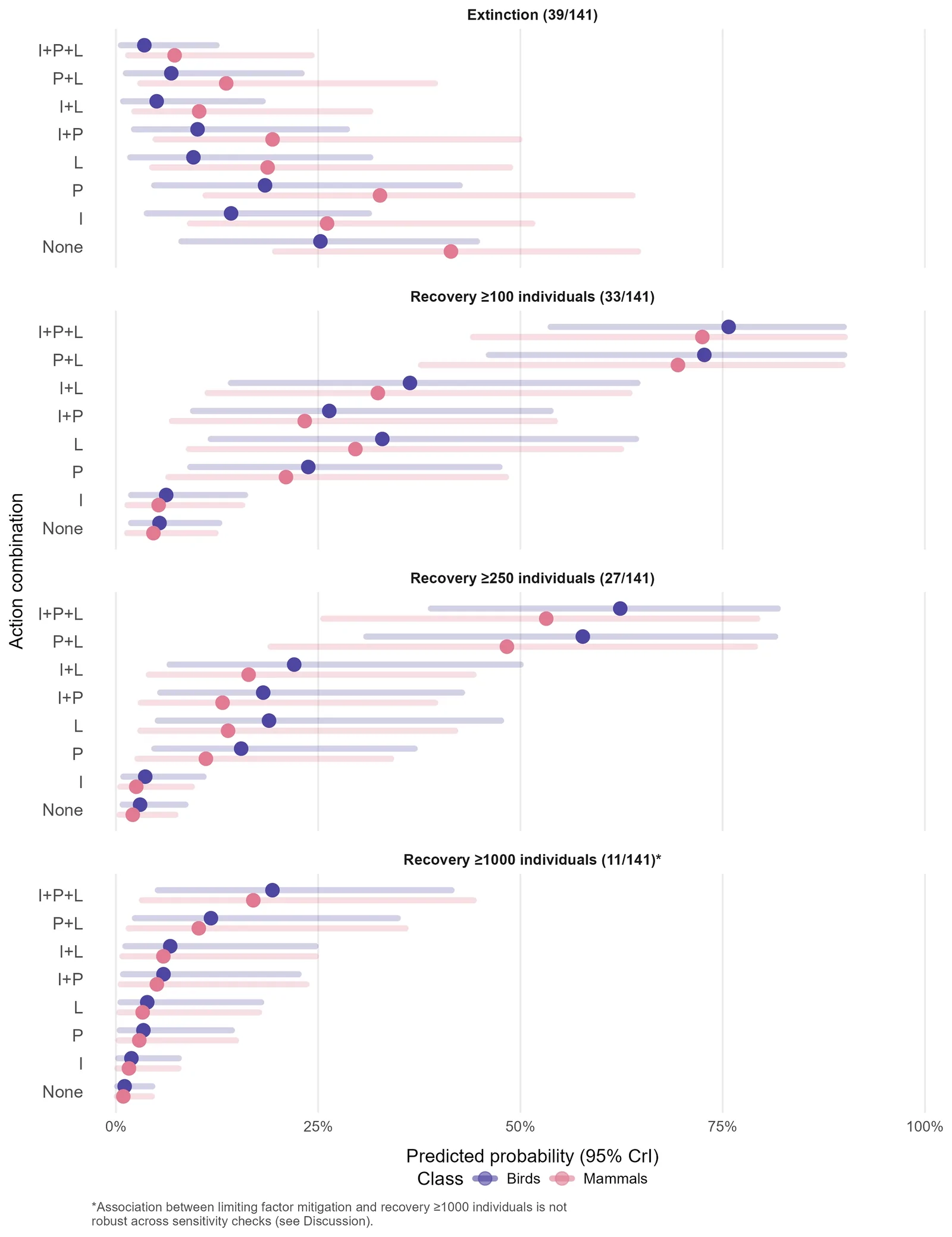
Posterior predicted probabilities of extinction and recovery for all eight combinations of indirect actions (I), limiting factor mitigation (L) and population manipulation (P), shown separately for birds and mammals. Points represent posterior medians and horizontal bars show 95% credible intervals. Predictions are population average values (marginal over taxonomic order) and are evaluated at the mean log-transformed generation length.

All three action classes trended towards reduced extinction risk, though all credible intervals overlapped one. Predicted probabilities nonetheless showed a clear ordering, from a median of 5% (birds) and 10% (mammals) with all action types combined, to 20% and 34%, respectively, with no recorded action. Taxonomic class affected baseline risk but not the direction of action effects. Mammals had higher extinction and lower recovery probabilities than birds under equivalent action combinations, but the relative benefit of both direct action types was consistent across classes and recovery levels, with substantially overlapping posterior intervals.

### (d) Sensitivity analyses

Every action–threshold combination for recovery ≥100 and ≥250 remained significant across every sensitivity check, including CR D non-action group thinning at 50% and both alternative priors (supplementary material, section S3). The association between limiting factor mitigation and recovery to ≥1000 was not robust, losing significance under exclusion of non-Red List sources, all three levels of CR D non-action group thinning, and under the tight prior. Adding threat burden as a covariate left the three action odds ratios essentially unchanged, though threat burden itself was a significant predictor in the expected direction. Indirect action's association with reduced extinction risk became formally significant once threat burden was included. The clearest movement came from the worst-case ‘possibly extinct’ recoding, under which all three action categories became significantly protective against extinction, a strengthening of the main finding.

## 4. Discussion

Our findings give a potent reminder of the ongoing tragedy of contemporary extinctions, with at least 39 species of birds and mammals that were extant in the middle of the twentieth century no longer with us 75 years later. But they also highlight a similarly sized group that has perhaps been less well appreciated as a collective: the 33 species that, having dropped below 50 individuals, managed to avoid extinction and reverse their decline to at least double in size to 100. That level remains critically low, but recovery in this group has predominantly been far stronger: 27 of these species now exceed 250 individuals and 11 exceed 1000. These recoveries are striking, and their apparent associations with direct conservation intervention are compelling: 61% of species that received direct action recovered to at least 100 individuals, compared with 2% of those that did not, and modelled odds of recovery to ≥100 and ≥250 were several times higher among species subject to direct action.

### (a) Applying the conservation toolkit

Conservationists have drawn on a diverse toolkit to confront the extrinsic factors limiting growth in small populations, with invasive species control, habitat restoration and the artificial provision of limited resources prominent. Direct population manipulation, particularly through *ex situ* care and translocation, has also been important. The combination of these two broad classes of action was associated with the highest recovery probabilities.

The case of the Lord Howe woodhen (*Hypotaenidia sylvestris*) displays the power of this combination. Endemic to Lord Howe Island, the once-abundant woodhen experienced a precipitous decline after the arrival of humans in 1788, driven by direct hunting combined with predation, competition and habitat degradation from introduced pigs, cats and rodents. By the late 1970s, an estimated 23 birds remained. In response, conservation efforts focused on the eradication of feral pigs and cats paired with a 4‑year captive breeding and release programme starting in 1980, with the population growing to at least 221 individuals by 2018 [51]. A 2019 rodent eradication posed a further risk through rodenticide bait exposure, and woodhens were again taken into temporary care for release after the baiting operation [51]. Post-release, after having spent four decades stable around 200 individuals, the population has rebounded strongly in a newly rodent-free habitat to an astonishing 1638 individuals by 2024.

### (b) The rarity of extinction once limiting factors are addressed

The Lord Howe woodhen's response to conservation action vividly illustrates the potential for recovery latent in some small populations. But what about the potential for small population effects to drive a continued descent to extinction, even after extrinsic factors limiting growth have been removed? Given that our dataset identifies small populations for which attempts have been made to address such limiting factors, in principle it should reveal candidates for extinction driven primarily by small population effects. In practice, it does not. Extinction in the presence of action to mitigate limiting factors is strikingly rare, occurring for just two species: the Aguijan reed-warbler (*Acrocephalus nijoi*) and the poʻouli. In neither case is there any suggestion that the drivers of decline were meaningfully alleviated before the species' demise [29,52], and so neither is a compelling example of the extinction vortex in action. It remains possible that such effects were material in our dataset but were offset by intensive population manipulation, whose role in avoiding extinction is unclear, perhaps because it is deployed in failed last-ditch efforts as well as successful ones. If that class of action has prevented small population effects from causing extinction, it is testament to how effectively conservationists have deployed their toolkit. Either way, Graeme Caughley's [5] observation that ‘no instance of extinction by genetic malfunction has been reported, whereas the examples of driven extinction are plentiful’ remains apt.

### (c) Long-term viability and conservation dependency

Our findings do not contradict the syntheses of population viability analyses arguing for minimum viable populations in the thousands. We chart the relatively short-term progress of managed species, whereas such analyses seek demographic targets for viability and evolutionary potential over longer periods, often centuries [4], and the deleterious impacts of severe bottlenecks may lag long after demographic recovery [53]. Nevertheless, many species here have shown ongoing recovery over a multi-decadal timeframe, over 50 years in several cases. The empirical record has delivered a flat rebuttal to predictions of the inevitability of extinction in the short term; longer-term claims are presently unfalsifiable. Indeed, there is considerable debate about generalized genetic extinction thresholds [7,54,55], with recognition that impacts on small populations can vary widely and warrant case-specific evaluation [56,57]. Recent assessments of the Hainan gibbon (*Nomascus hainanus*) and vaquita (*Phocoena sinus*), with populations of 45 individuals and 10 individuals, respectively, found that neither has experienced substantial inbreeding depression [24,58,59], and limited genome-wide consequences of severe bottlenecks have been shown for other recovered populations [60,61]. More ancient bottlenecks have had ongoing deleterious effects on modern resilience [62], but many species have been able to recover from catastrophic bottlenecks in their evolutionary history [63,64], indicating the potential for long-term viability following recovery if limiting factors are mitigated.

Continued recovery may entail continued investment. Although some species, such as the Rodrigues fody (*Foudia flavicans*), Rodrigues warbler (*Acrocephalus rodericanus*) and Seychelles kestrel (*Falco araeus*), have recovered with minimal ongoing species-specific efforts [65], many other ‘recovered’ species remain intensively managed. Island invasive species eradication typically requires long-term biosecurity effort [66]; megafaunal mammals may require continued protection from poaching long after a crash [67]; and many recoveries involve sustained captive breeding, with *ex situ* insurance populations retained after wild recovery [68]. Small populations are typically associated with longer-term conservation dependence [35], requiring financial resources and applied skills over decades. Our findings suggest that such investment is associated with continued progress rather than mere maintenance and need not be regarded as futile or inappropriate.

### (d) Interpreting association

This study charts a diverse, uneven and non-random allocation of conservation efforts spanning 141 species across 75 years. We do not present the findings of a controlled experiment from which the outcome can be unambiguously attributed to the treatment, and so the associations we report could arise in ways other than action promoting recovery. The extreme alternative—that recovery drives action, with conservationists attaching themselves to populations already increasing for other reasons—would require drivers of severe decline to abate independently of and prior to intervention. This is testable in part with our timing data, which instead show that action tended to start when species were in decline, with the drop below 50 happening either after action initiation (41% of cases (20 of 49) where timing of both is recorded) or soon before (within the longer of three generations or 10 years prior, 39% of cases) in the majority of cases, with no association between this timing gap and recovery (Kruskal–Wallis *p* = 0.29).

A more serious possibility is that action was directed at more tractable problems, in which case our recovery rates would overstate what expanded conservation effort could achieve, since expansion would mean moving into harder cases. A cross-check of Red List threat categories causing ongoing or historical significant declines or worse over the majority of a species' population does not indicate selectivity in action at this level, with no threat category showing a significant association with either direct action or recovery (Fisher's exact test on species-level 2 × 2 tables, with Benjamini-Hochberg correction within each outcome: supplementary material, section S4). Nevertheless, tractability bias is plausible: the conservation toolbox is not yet equipped with well-established remedies for all challenges. Species that are impacted by the presence of invasive rats, for example, can benefit from proven rodent control and eradication techniques [69], but management of threats such as avian malaria affecting forest birds in Hawaii remains extremely challenging [70]. Species themselves can vary in their inherent amenability to conservation management techniques such as *ex situ* conservation efforts [71,72], with capture of founder individuals and captive breeding less feasible for some highly threatened species owing to both ecological factors and available resources [73]. Ultimately, whether challenging contexts have led to inaction in some species, our review of the population statuses of those that *have* been subject to action points to a strong recovery potential in small populations and underscores the value and urgency in continuing to develop solutions to harder problems for more species.

We note finally that, other than for the baiji [23], we encountered little explicit evidence of triage leading to intentional inaction and subsequent extinction. Conservationists were aware of the plight of the Christmas Island pipistrelle (*Pipistrellus murrayi*) for over a decade before its extinction, but the absence of intervention arose from indecision and delay rather than a deliberate choice [74]. Similar decision paralysis affected the extinct poʻouli [75], and practitioners have struggled to act effectively for the near-extinct vaquita [24]. These species are nonetheless conservatively recorded in our dataset as having been subject to direct action. Imminent extinction can even act to attract conservation action: precipitous declines in the Guam flycatcher (*Myiagra freycineti*), probably driven by the introduction of the brown tree snake (*Boiga irregularis*), led to attempts to capture remaining individuals for *ex situ* management, with one male caught in 1983 dying in captivity a year later, just months before the last individual of the species was seen in the wild [76].

### (e) Limitations

Care must be taken to reflect on the limitations inherent to a study such as this. Though our scope—bird and mammal species that have been extant but below 50 individuals at any point since 1950—is itself simple and explicit, the historical documentation is heterogeneous in nature and quality. To what extent might we have left out species that met the criteria, or included species that did not? It is important to note that a species could not have been omitted merely through oversight: all birds and 88% of mammals have been evaluated for the Red List, which acts as a de facto first filter for this study. Erroneous exclusion would require an assessment to either incorrectly list a qualifying species as extinct before 1950, to incorrectly place a population above 50, or for an unrecorded dip and recovery to have occurred. Population overestimation owing to assessment lag is plausible, but our exclusion of species whose most recent assessment falls within the longer of three generations or 10 years of a dip below 50 buffers against this.

Red List assessments may precautionarily but incorrectly place poorly documented species—which are also those unlikely to receive direct action—below 50 mature individuals. This would act to artificially inflate the group coded as below 50 receiving no action and overstate the difference action makes. Targeted correction is difficult for exactly the reason the problem arises, but the association between direct action and recovery to ≥100 and ≥250 remained robust to random removal of up to 50% of CR D species lacking direct action (supplementary material, table S3c).

Brief or unsuccessful conservation interventions could go unreported, but we analysed action at a higher level than it was recorded, meaning that most classifications are supported by multiple distinct activities, giving redundancy against under-reporting. For undocumented action to meaningfully affect our findings, it would need to lead to a species being misclassified as receiving no intervention whatsoever within our broad categories. Further, our conservative coding counts even minor or failed attempts as direct action, including for species that subsequently went extinct.

Population estimates varied in method, quality and certainty, but our classes—aligned with Red List bands—are deliberately crude to accommodate this. Following the Red List, we used the minimum bound of any estimate when assigning classes. No estimate range straddled a class boundary, and most species sit well above their class minimum (100–249: median 165, no species within 20% of the bound; 250–999: median 369, 13% within 20%; ≥1000: median 3000, 9% within 20%). Where timing of action and population threshold crossing was uncertain, we used range midpoints and tested sensitivity to action misclassification by rerunning models at opposing timing extremes: action dates at their earliest bound and population threshold-crossing dates at their latest bound, and the reverse. Reclassification was rare—at most one species per action category and threshold—producing no material change to any model (supplementary material, table S3f).

### (f) Conclusion

The extinction crisis continued apace during the preparation of this study, with avian malaria-driven declines in two Hawaiian honeycreepers, the ʻakekeʻe (*Loxops caeruleirostris*) and the ʻakikiki (*Oreomystis bairdi*), sufficiently severe as to tragically render both species imminent candidates for our dataset. The ʻakikiki, whose current (version 2025-2) Red List assessment estimates 468 individuals [77], is facing extinction in the wild, having since declined to an estimated four non-captive individuals as of 2024 [78], with a captive breeding programme established in 2015 currently securing the species [79]. The ʻakekeʻe, whose current Red List assessment estimates 945 individuals [80], has since dropped to fewer than 100 [81], with unsuccessful attempts to start a captive breeding programme leaving its future dependent on tackling avian malaria [81]. Recent genomics-informed population modelling predicts that the ʻakekeʻe can evade extinction and recover if this threat can be effectively controlled [81], aligning with the broader recovery record documented here.

Predictions of extinction as an inevitable fate may be self-fulfilling, and we are probably fortunate that countless determined conservationists did not view their situations this way, and instead undertook bold interventions to confront threats and recover species on the brink. Their efforts combine to deliver an empirically grounded note of hope that challenges more pessimistic prognoses based on contestable interpretations of theory, and reaffirms the urgent need for decisive action.

## Supplementary Material



## Data Availability

All data and code used to generate the findings in the paper are provided in the supplementary material. Supplementary material is available online [82].
