# Supplementary material for: Inevitable fates? The recovery potential of small populations
Source: Proc Biol Sci. 2026 Nov 4;293(2082):20262436. doi: 10.1098/rspb.2026.2436 (PMC13730801; doi:10.1098/rspb.2026.2436)
Supplement: Supplementary file 1 [file rspb.2026.2436.zip › rspb.2026.2436/rspb20262436_si_001.pdf]

SUPPLEMENTARY MATERIAL TO RSPB-2026-2436

Title: Inevitable fates? The recovery potential of small populations

Authors: Donal Smith[1,2]*, John G. Ewen[1], Rohan H. Clarke[2], Zarah Wessels[2], Samuel T. Turvey[1]

[1]*Institute of Zoology, Zoological Society of London, Regent's Park, London, UK*

[2]*School of Biological Sciences, Monash University, Clayton, Victoria, Australia*

Journal: Proceedings B

DOI: 10.1098/rspb.2026.2436

S1. Species excluded from the study

Table S1a. Species screened out of study on the basis of insufficient evidence to support their being extant and below 50 individuals at any point after 1950.

Scientific name	Common name	Class	Inclusion criteria	Exclusion criteria	Comments
Dendrolagus mayri	Wondiwoi Tree-kangaroo	Mammals	CR D	Red List [1] superseded	Listed as Possibly Extinct on Red List but this assessment was prior to it being photographed in 2018. One report states "the Wondiwoi tree kangaroo has a very limited distribution— perhaps just 40 to 80 square miles—but the numerous scratch marks and dung suggest "it is amazingly common in a very small area," [2]. Thus no reason to conclude species was ever below 50.
Dipodomys gravipes	San Quintin Kangaroo Rat	Mammals	CR D	Red List superseded	Red List assessment (made in 2016) is superseded by rediscovery of species in 2017 and subsequent surveys that show the population is at least above 50. E.g. "We captured a total of 83 San Quintín kangaroo rats at 13 sites within and 6 beyond the known distribution" [3]
Fregetta maoriana	New Zealand Storm-petrel	Birds	CR D	Red List superseded	Though most recent assessment (2018) places the New Zealand Storm-petrel in CR D, recent mark-capture studies [4] favour an estimation of a current population of 1 630 individuals (range between two models 446 – 3 758) with an estimated low point prior to kiore eradication on Hauturu of between 300 and 800. Though cat and kiore eradications on Hauturu may have saved the species from extinction, there does not appear to be any support for the idea that the population ever dropped below 50 individuals.
Lagorchestes asomatus	Kuluwarri	Mammals	EX post 1950	Last confirmed record pre-1950	Red List last seen field lists "1950s", but narrative lists last confirmed sighting in 1932.
Lepilemur septentrionalis	Sahafary Sportive Lemur	Mammals	CR D	Red List superseded	Recent population estimate (87) represents increase on previous estimates [5]. The authors speculate that the apparent increase may be "artificial due to the loss and fragmentation of their forest, causing the lemurs to be more densely populated in the remaining forest patches." In any case, the previous estimate (Red List) was approx. 50-70. Taken together, there is no clear claim of the population having been below 50.
Nesospiza wilkinsi	Wilkins's Finch	Birds	CR D	Red List superseded	Red List assessment (2021) appears to be a precautionary placement below 50 based on incomplete surveys following a destructive storm. Follow-up surveys [6] suggest that the damage to the population (which was in 2024 estimated to number 60 - 90

					pairs) was less severe than previously thought. For these reasons the species is not thought to be a credible example of having been below 50 individuals.
Nomascus nasutus	Cao-vit Gibbon	Mammals	CR D	Red List error - population size	Category D and population size from Red List appear to be an error. Narrative text cites >129 individuals from most recent (and only partial) survey
Notamacropus greyi	Toolache Wallaby	Mammals	EX post 1950	Red List error - last seen	Year last seen field in Red List assessment (entered as "1970s") appears to be an error. From Red List narrative text: "the last confirmed records were in 1924", "last record in captivity in 1939", "The estimated extinction date based on confirmed sightings was 1932"
Nyctophilus howensis	Lord Howe Long-eared Bat	Mammals	EX post 1950	Red List error- last seen	Year last seen field in Red List assessment (1972) appears to refer to the year in which a skull, the only record of the species, was discovered. The skull is dated approximately to the 20th century. As there is no live record past 1950, the species is excluded.
Thalasseus bernsteini	Chinese Crested Tern	Birds	CR D	Red List superseded	Total population size poorly known and generally regarded on the Red List as below 50. However, more recent work [7] reports counts across just two sites exceeding 70 breeding individuals and suggesting the presence of previously-unidentified breeding sites. There in insufficient evidence to conclude that this species was ever below 50 individuals.

Table S1b. Species excluded on the basis of having dropped below 50 within the longer of three generations or 10 years of their most recent assessment or population estimate.

Scientific name	Common name	Class	Generation length	Generation-calibrated time window (Longer of generation length x3 or 10 years)	Estimated year below 50	Year eligible for inclusion in analysis	Year of recent population estimate
Dicerorhinus sumatrensis	Sumatran Rhinoceros	Mammals	20	60	2020	2080	2024

Lipotes vexillifer	Baiji	Mammals	13.27	40	1998	2038	2017
Phocoena sinus	Vaquita	Mammals	10	30	2016	2046	2018
Pomarea whitneyi	Fatu Hiva Monarch	Birds	6.9	21	2010	2031	2024
Sitta insularis	Bahama Nuthatch	Birds	2.23	10	2016	2026	2024
Trachypithecus poliocephalus	Cat Ba Hooded Black Leaf Monkey	Mammals	11	33	2000	2033	2023

S2. Species recovered to 100 individuals or more

Wild population low points and recent population estimates for mammal and bird species that, having been below 50 individuals in the wild at any point after 1950, have since recovered to populations of 100 individuals or more.

Scientific name	Common name	Class	Wild population low point (and years)	Recent wild population estimate (year)	References
Dama mesopotamica	Persian Fallow Deer	Mammals	Unknown (>8 *ex situ* in 1957–1965)	>380 (2025)	[1,8]
Bison bonasus	European Bison	Mammals	0 (12 *ex situ* before 1927)	6 244 (2019)	[1,9,10]
Mustela nigripes	Black-footed Ferret	Mammals	0 (15 *ex situ* in 1987)	340 (2019)	[9,11,12]
Oryx leucoryx	Arabian Oryx	Mammals	0 (17 *ex situ* in 1960s)	1 220 (2016)	[1,9,13]

Elaphurus davidianus	Père David's Deer	Mammals	0 (18 *ex situ* in 1900–1910)	2 825 (2021)	[14,15]
Gymnogyps californianus	California Condor	Birds	0 (22 *ex situ* in 1982)	369 (2024)	[16,17]
Equus ferus	Przewalski's Horse	Mammals	0 (31 *ex situ* in 1940s)	387 (2014)	[1,18]
Oryx dammah	Scimitar-horned Oryx	Mammals	0 (40–50 *ex situ* in 1960s)	600 (2023)	[1,19]
Falco punctatus	Mauritius Kestrel	Birds	4 (1974)	<250 (2021)	[1,20]
Nipponia nippon	Asian Crested Ibis	Birds	7 (1981)	1 022 (2019)	[21,22]
Petroica traversi	Black Robin	Birds	5 (1979–1980)	350 (2025)	[1,23]
Nesoenas mayeri	Pink Pigeon	Birds	9-10 (1990)	308 (2020)	[1,24]
Foudia flavicans	Rodrigues Fody	Birds	10-12 (1968)	20 000 (2016)	[1,25]
Copsychus sechellarum	Seychelles Magpie-robin	Birds	12 (1965)	400 (2021)	[26,27]
Amazona vittata	Puerto Rican Amazon	Birds	13 (1975)	260 (2022)	[1,28]
Potorous gilbertii	Gilbert's Potoroo	Mammals	<20 (before 1994)	103 (2018)	[29,30]
Alexandrinus eques	Echo Parakeet	Birds	>20 (1980–1982)	756 (2018)	[1,31]
Grus americana	Whooping Crane	Birds	21 (1955)	698 (2024)	[32,33]

Hypotaenidia sylvestris	Lord Howe Woodhen	Birds	23 (1970s)	1 638 (2024)	[34,35]
Phoebastria albatrus	Short-tailed albatross	Birds	23 (1954)	4 200 (2014)	[1,36]
Himantopus novaezelandiae	Black Stilt	Birds	28 (1980)	156 (2023)	[1,37]
Pomarea dimidiata	Rarotonga Monarch	Birds	29 (1989)	600 (2020)	[38,39]
Marmota vancouverensis	Vancouver Island Marmot	Mammals	30 (2003)	140–190 (2016)	[40,41]
Acrocephalus rodericanus	Rodrigues Warbler	Birds	<30 (1968–1974)	25 000 (2016)	[1,42]
Acrocephalus sechellensis	Seychelles Warbler	Birds	<30 (1959–1968)	3 000 (2016)	[1,43]
Falco araeus	Seychelles Kestrel	Birds	<30 (1940s–1960s)	700–900 (2023)	[1,44]
Branta sandvicensis	Hawaiian Goose	Birds	~ 33 (1951–1960)	2 600–3 300 (2018)	[45,46]
Pterodroma cahow	Bermuda Petrel	Birds	~ 34 (1963–1964)	330 (2025)	[47,48]
Pomarea nigra	Ōmāma'o; Tahiti Monarch	Birds	<36 (2002–2009)	151 (2025)	[49,50]
Philesturnus carunculatus	South Island Saddleback	Birds	36 (1962)	5 000 (2020)	[1,51]
Lasiorhinus krefftii	Northern Hairy-nosed Wombat	Mammals	30–40 (1960–1982)	315 (2021)	[52,53]

Atlapetes pallidiceps	Pale-headed Brush-finch	Birds	~ 40 (1999)	~ 200 (2020)	[1,54]
Anas nesiotis	Campbell Teal	Birds	<50 (before 1999)	500–1 000 (2020)	[1,55]

S3. Full sensitivity analysis results

Table S3a. Prior sensitivity.

Primary models refit under 'tight' (normal(0, 0.5)) and 'wide' (normal(0, 2)) priors on the log-odds scale, vs. default normal(0, 1).

Model	Term	OR
**Extinct (default)**	Intercept	0.32 (0.09–0.81)
**Extinct (default)**	Indirect action	0.50 (0.21–1.13)
**Extinct (default)**	Limiting factor mitigation	0.32 (0.10–1.02)
**Extinct (default)**	Population manipulation	0.68 (0.25–1.85)
**Extinct (default)**	Generation length (per 1 SD in log)	0.60 (0.35–1.02)
**Extinct (default)**	Class: Mammals	2.19 (0.72–7.91)
**Extinct (tight)**	Intercept	0.35 (0.13–0.74)
**Extinct (tight)**	Indirect action	0.59 (0.31–1.13)
**Extinct (tight)**	Limiting factor mitigation	0.53 (0.25–1.14)
**Extinct (tight)**	Population manipulation	0.73 (0.36–1.48)
**Extinct (tight)**	Generation length (per 1 SD in log)	0.63 (0.40–1.00)

**Extinct (tight)**	Class: Mammals	1.47 (0.69–3.27)
**Extinct (wide)**	Intercept	0.21 (0.03–0.73)
**Extinct (wide)**	Indirect action	0.48 (0.18–1.22)
**Extinct (wide)**	Limiting factor mitigation	0.19 (0.03–0.85)
**Extinct (wide)**	Population manipulation	0.73 (0.20–2.59)
**Extinct (wide)**	Generation length (per 1 SD in log)	0.60 (0.33–1.08)
**Extinct (wide)**	Class: Mammals	4.13 (0.85–31.92)
**Recovery ≥100 (default)**	Intercept	0.06 (0.02–0.15)
**Recovery ≥100 (default)**	Indirect action	1.15 (0.41–3.16)
**Recovery ≥100 (default)**	Limiting factor mitigation	8.73 (3.09–25.63)
**Recovery ≥100 (default)**	Population manipulation	5.53 (2.09–14.84)
**Recovery ≥100 (default)**	Generation length (per 1 SD in log)	1.42 (0.82–2.47)
**Recovery ≥100 (default)**	Class: Mammals	0.85 (0.28–2.52)
**Recovery ≥100 (tight)**	Intercept	0.10 (0.04–0.20)
**Recovery ≥100 (tight)**	Indirect action	1.30 (0.65–2.66)
**Recovery ≥100 (tight)**	Limiting factor mitigation	3.99 (1.99–8.05)
**Recovery ≥100 (tight)**	Population manipulation	3.28 (1.63–6.60)
**Recovery ≥100 (tight)**	Generation length (per 1 SD in log)	1.43 (0.93–2.26)

**Recovery ≥100 (tight)**	Class: Mammals	0.91 (0.43–1.85)
**Recovery ≥100 (wide)**	Intercept	0.04 (0.01–0.12)
**Recovery ≥100 (wide)**	Indirect action	0.90 (0.24–3.07)
**Recovery ≥100 (wide)**	Limiting factor mitigation	17.33 (4.76–79.49)
**Recovery ≥100 (wide)**	Population manipulation	7.94 (2.47–27.12)
**Recovery ≥100 (wide)**	Generation length (per 1 SD in log)	1.40 (0.73–2.66)
**Recovery ≥100 (wide)**	Class: Mammals	0.73 (0.14–2.99)
**Recovery ≥250 (default)**	Intercept	0.03 (0.01–0.09)
**Recovery ≥250 (default)**	Indirect action	1.21 (0.42–3.48)
**Recovery ≥250 (default)**	Limiting factor mitigation	7.65 (2.51–24.83)
**Recovery ≥250 (default)**	Population manipulation	5.97 (2.03–18.41)
**Recovery ≥250 (default)**	Generation length (per 1 SD in log)	1.50 (0.82–2.77)
**Recovery ≥250 (default)**	Class: Mammals	0.69 (0.22–2.12)
**Recovery ≥250 (tight)**	Intercept	0.06 (0.03–0.14)
**Recovery ≥250 (tight)**	Indirect action	1.29 (0.64–2.66)
**Recovery ≥250 (tight)**	Limiting factor mitigation	3.61 (1.75–7.72)
**Recovery ≥250 (tight)**	Population manipulation	3.31 (1.58–6.82)
**Recovery ≥250 (tight)**	Generation length (per 1 SD in log)	1.49 (0.92–2.45)

**Recovery ≥250 (tight)**	Class: Mammals	0.81 (0.38–1.75)
**Recovery ≥250 (wide)**	Intercept	0.02 (0.00–0.07)
**Recovery ≥250 (wide)**	Indirect action	1.05 (0.27–3.87)
**Recovery ≥250 (wide)**	Limiting factor mitigation	14.64 (3.33–74.29)
**Recovery ≥250 (wide)**	Population manipulation	9.17 (2.24–40.77)
**Recovery ≥250 (wide)**	Generation length (per 1 SD in log)	1.51 (0.75–3.10)
**Recovery ≥250 (wide)**	Class: Mammals	0.58 (0.11–2.48)
**Recovery ≥1000 (default)**	Intercept	0.01 (0.00–0.05)
**Recovery ≥1000 (default)**	Indirect action	1.79 (0.52–6.32)
**Recovery ≥1000 (default)**	Limiting factor mitigation	3.84 (1.01–15.86)
**Recovery ≥1000 (default)**	Population manipulation	3.28 (0.87–12.92)
**Recovery ≥1000 (default)**	Generation length (per 1 SD in log)	1.30 (0.63–2.67)
**Recovery ≥1000 (default)**	Class: Mammals	0.85 (0.21–3.03)
**Recovery ≥1000 (tight)**	Intercept	0.03 (0.01–0.08)
**Recovery ≥1000 (tight)**	Indirect action	1.46 (0.65–3.29)
**Recovery ≥1000 (tight)**	Limiting factor mitigation	2.12 (0.92–4.92)
**Recovery ≥1000 (tight)**	Population manipulation	2.00 (0.86–4.46)
**Recovery ≥1000 (tight)**	Generation length (per 1 SD in log)	1.33 (0.75–2.35)

**Recovery ≥1000 (tight)**	Class: Mammals	0.93 (0.40–2.09)
**Recovery ≥1000 (wide)**	Intercept	0.00 (0.00–0.03)
**Recovery ≥1000 (wide)**	Indirect action	2.15 (0.44–11.81)
**Recovery ≥1000 (wide)**	Limiting factor mitigation	7.53 (1.14–68.61)
**Recovery ≥1000 (wide)**	Population manipulation	5.21 (0.78–44.80)
**Recovery ≥1000 (wide)**	Generation length (per 1 SD in log)	1.25 (0.49–2.92)
**Recovery ≥1000 (wide)**	Class: Mammals	0.71 (0.08–4.73)

Table S3b. Source of inclusion outside Red List framework.

Refit models excluding species identified via non Red List related sources (n=13: *Acrocephalus sechellensis, Branta sandvicensis, Dama mesopotamica, Falco araeus, Foudia flavicans, Grus americana, Hypotaenidia sylvestris, Lasiorhinus krefftii, Marmota vancouverensis, Petroica traversi, Philesturnus carunculatus, Phoebastria albatrus, Pterodroma cahow*.).

Model	Species set	Term	OR
**Extinction**	All 141	Indirect action	0.50 (0.21–1.13)
**Extinction**	Excl. non-Red-List	Indirect action	0.47 (0.19–1.11)
**Extinction**	All 141	Limiting factor mitigation	0.32 (0.10–1.02)
**Extinction**	Excl. non-Red-List	Limiting factor mitigation	0.33 (0.09–1.12)
**Extinction**	All 141	Population manipulation	0.68 (0.25–1.85)
**Extinction**	Excl. non-Red-List	Population manipulation	0.90 (0.30–2.76)

**Recovery ≥100**	All 141	Indirect action	1.16 (0.41–3.16)
**Recovery ≥100**	Excl. non-Red-List	Indirect action	1.46 (0.47–4.55)
**Recovery ≥100**	All 141	Limiting factor mitigation	8.71 (3.09–25.63)
**Recovery ≥100**	Excl. non-Red-List	Limiting factor mitigation	10.35 (3.06–37.08)
**Recovery ≥100**	All 141	Population manipulation	5.53 (2.09–14.84)
**Recovery ≥100**	Excl. non-Red-List	Population manipulation	5.57 (1.81–18.40)
**Recovery ≥1000**	All 141	Indirect action	1.78 (0.52–6.32)
**Recovery ≥1000**	Excl. non-Red-List	Indirect action	2.63 (0.60–11.85)
**Recovery ≥1000**	All 141	Limiting factor mitigation	3.81 (1.01–15.86)
**Recovery ≥1000**	Excl. non-Red-List	Limiting factor mitigation	2.26 (0.53–10.39)
**Recovery ≥1000**	All 141	Population manipulation	3.23 (0.87–12.92)
**Recovery ≥1000**	Excl. non-Red-List	Population manipulation	2.13 (0.47–9.70)
**Recovery ≥250**	All 141	Indirect action	1.22 (0.42–3.48)
**Recovery ≥250**	Excl. non-Red-List	Indirect action	1.72 (0.50–6.08)
**Recovery ≥250**	All 141	Limiting factor mitigation	7.56 (2.51–24.83)
**Recovery ≥250**	Excl. non-Red-List	Limiting factor mitigation	6.90 (1.93–26.66)
**Recovery ≥250**	All 141	Population manipulation	5.94 (2.03–18.41)
**Recovery ≥250**	Excl. non-Red-List	Population manipulation	6.56 (1.92–23.95)

Table S3c. Thinning of CR D species with no direct action.

Refit models with the no-direct-action CR D group reduced by random removal at 10%, 25%, and 50%

Model	Percent removed	Term	OR
**Extinction**	0	Indirect action	0.50 (0.21–1.13)
**Extinction**	10	Indirect action	0.49 (0.20–1.16)
**Extinction**	25	Indirect action	0.39 (0.16–0.92)
**Extinction**	50	Indirect action	0.43 (0.17–1.06)
**Extinction**	0	Limiting factor mitigation	0.32 (0.10–1.02)
**Extinction**	10	Limiting factor mitigation	0.31 (0.09–0.98)
**Extinction**	25	Limiting factor mitigation	0.29 (0.09–0.94)
**Extinction**	50	Limiting factor mitigation	0.26 (0.08–0.82)
**Extinction**	0	Population manipulation	0.68 (0.25–1.85)
**Extinction**	10	Population manipulation	0.66 (0.23–1.83)
**Extinction**	25	Population manipulation	0.55 (0.19–1.53)
**Extinction**	50	Population manipulation	0.45 (0.16–1.26)
**Recovery ≥100**	0	Indirect action	1.16 (0.41–3.16)
**Recovery ≥100**	10	Indirect action	1.16 (0.42–3.15)
**Recovery ≥100**	25	Indirect action	1.09 (0.40–2.91)

**Recovery ≥100**	50	Indirect action	1.14 (0.41–3.09)
**Recovery ≥100**	0	Limiting factor mitigation	8.71 (3.09–25.63)
**Recovery ≥100**	10	Limiting factor mitigation	8.37 (3.00–23.92)
**Recovery ≥100**	25	Limiting factor mitigation	8.17 (3.04–23.90)
**Recovery ≥100**	50	Limiting factor mitigation	7.42 (2.65–22.10)
**Recovery ≥100**	0	Population manipulation	5.53 (2.09–14.84)
**Recovery ≥100**	10	Population manipulation	5.44 (2.04–14.44)
**Recovery ≥100**	25	Population manipulation	5.15 (2.00–13.62)
**Recovery ≥100**	50	Population manipulation	4.60 (1.74–12.32)
**Recovery ≥1000**	0	Indirect action	1.78 (0.52–6.32)
**Recovery ≥1000**	10	Indirect action	1.81 (0.53–6.88)
**Recovery ≥1000**	25	Indirect action	1.78 (0.51–6.49)
**Recovery ≥1000**	50	Indirect action	1.76 (0.53–6.49)
**Recovery ≥1000**	0	Limiting factor mitigation	3.81 (1.01–15.86)
**Recovery ≥1000**	10	Limiting factor mitigation	3.71 (0.99–14.74)
**Recovery ≥1000**	25	Limiting factor mitigation	3.52 (0.93–14.96)
**Recovery ≥1000**	50	Limiting factor mitigation	3.46 (0.94–13.53)
**Recovery ≥1000**	0	Population manipulation	3.23 (0.87–12.92)

**Recovery ≥1000**	10	Population manipulation	3.28 (0.89–12.78)
**Recovery ≥1000**	25	Population manipulation	3.03 (0.78–12.71)
**Recovery ≥1000**	50	Population manipulation	2.90 (0.80–11.74)
**Recovery ≥250**	0	Indirect action	1.22 (0.42–3.48)
**Recovery ≥250**	10	Indirect action	1.20 (0.42–3.38)
**Recovery ≥250**	25	Indirect action	1.19 (0.41–3.25)
**Recovery ≥250**	50	Indirect action	1.15 (0.40–3.33)
**Recovery ≥250**	0	Limiting factor mitigation	7.56 (2.51–24.83)
**Recovery ≥250**	10	Limiting factor mitigation	7.42 (2.50–24.32)
**Recovery ≥250**	25	Limiting factor mitigation	7.17 (2.42–22.79)
**Recovery ≥250**	50	Limiting factor mitigation	6.94 (2.34–21.66)
**Recovery ≥250**	0	Population manipulation	5.94 (2.03–18.41)
**Recovery ≥250**	10	Population manipulation	5.78 (2.01–18.03)
**Recovery ≥250**	25	Population manipulation	5.51 (1.81–16.97)
**Recovery ≥250**	50	Population manipulation	5.16 (1.72–16.30)

Table S3d. Possibly Extinct recoding.

Refit extinction model recoding all Possibly Extinct species (n=22) as Extinct (worst-case scenario).

Scenario	Term	OR
**Possibly Extinct as extant**	Indirect action	0.50 (0.21–1.13)
**Possibly Extinct as extant**	Limiting factor mitigation	0.32 (0.10–1.02)
**Possibly Extinct as extant**	Population manipulation	0.68 (0.25–1.85)
**Possibly Extinct as extinct**	Indirect action	0.28 (0.13–0.61)
**Possibly Extinct as extinct**	Limiting factor mitigation	0.20 (0.06–0.59)
**Possibly Extinct as extinct**	Population manipulation	0.32 (0.12–0.81)

Table S3e. Red List threat category inclusion

Refit models with added covariate: count of distinct ongoing or historical threats causing 'slow, significant declines' or worse over the majority of the population listed in Red List assessments

Model	Threat covariate	Term	OR
**Extinction**	With	Indirect action	0.40 (0.16–0.98)
**Extinction**	Without	Indirect action	0.50 (0.21–1.13)
**Extinction**	With	Limiting factor mitigation	0.35 (0.09–1.15)
**Extinction**	Without	Limiting factor mitigation	0.32 (0.10–1.02)
**Extinction**	With	Population manipulation	0.68 (0.22–1.91)
**Extinction**	Without	Population manipulation	0.68 (0.25–1.85)
**Recovery ≥100**	With	Indirect action	1.33 (0.48–3.61)

**Recovery ≥100**	Without	Indirect action	1.16 (0.41–3.16)
**Recovery ≥100**	With	Limiting factor mitigation	9.08 (3.27–26.92)
**Recovery ≥100**	Without	Limiting factor mitigation	8.71 (3.09–25.63)
**Recovery ≥100**	With	Population manipulation	6.45 (2.36–18.47)
**Recovery ≥100**	Without	Population manipulation	5.53 (2.09–14.84)
**Recovery ≥1000**	With	Indirect action	2.03 (0.59–6.91)
**Recovery ≥1000**	Without	Indirect action	1.78 (0.52–6.32)
**Recovery ≥1000**	With	Limiting factor mitigation	3.87 (0.99–15.20)
**Recovery ≥1000**	Without	Limiting factor mitigation	3.81 (1.01–15.86)
**Recovery ≥1000**	With	Population manipulation	3.43 (0.90–13.14)
**Recovery ≥1000**	Without	Population manipulation	3.23 (0.87–12.92)
**Recovery ≥250**	With	Indirect action	1.37 (0.48–4.08)
**Recovery ≥250**	Without	Indirect action	1.22 (0.42–3.48)
**Recovery ≥250**	With	Limiting factor mitigation	8.26 (2.74–26.66)
**Recovery ≥250**	Without	Limiting factor mitigation	7.56 (2.51–24.83)
**Recovery ≥250**	With	Population manipulation	6.67 (2.20–20.67)
**Recovery ≥250**	Without	Population manipulation	5.94 (2.03–18.41)

Table S3f. Action and population timings variation.

Refit under two extremes that maximise the chance of an action reclassification: action dates to their earliest bound and population threshold-crossing dates to their latest bound, and the reverse.

Model	Timing scenario	Term	OR
**Extinction**	Default (time range midpoints)	Indirect action	0.50 (0.21–1.13)
**Extinction**	Earliest possible action, latest possible threshold crossing	Indirect action	0.49 (0.21–1.12)
**Extinction**	Latest possible action, earliest possible threshold crossing	Indirect action	0.48 (0.20–1.12)
**Extinction**	Default (time range midpoints)	Limiting factor mitigation	0.32 (0.10–1.02)
**Extinction**	Earliest possible action, latest possible threshold crossing	Limiting factor mitigation	0.31 (0.09–1.00)
**Extinction**	Latest possible action, earliest possible threshold crossing	Limiting factor mitigation	0.35 (0.10–1.13)
**Extinction**	Default (time range midpoints)	Population manipulation	0.68 (0.25–1.85)
**Extinction**	Earliest possible action, latest possible threshold crossing	Population manipulation	0.71 (0.25–1.97)
**Extinction**	Latest possible action, earliest possible threshold crossing	Population manipulation	0.68 (0.24–1.84)
**Recovery ≥100**	Default (time range midpoints)	Indirect action	1.16 (0.41–3.16)
**Recovery ≥100**	Earliest possible action, latest possible threshold crossing	Indirect action	1.19 (0.44–3.14)
**Recovery ≥100**	Latest possible action, earliest possible threshold crossing	Indirect action	1.26 (0.46–3.31)
**Recovery ≥100**	Default (time range midpoints)	Limiting factor mitigation	8.71 (3.09–25.63)
**Recovery ≥100**	Earliest possible action, latest possible threshold crossing	Limiting factor mitigation	9.84 (3.57–29.28)
**Recovery ≥100**	Latest possible action, earliest possible threshold crossing	Limiting factor mitigation	6.87 (2.53–19.77)

**Recovery ≥100**	Default (time range midpoints)	Population manipulation	5.53 (2.09–14.84)
**Recovery ≥100**	Earliest possible action, latest possible threshold crossing	Population manipulation	5.20 (1.98–13.49)
**Recovery ≥100**	Latest possible action, earliest possible threshold crossing	Population manipulation	5.71 (2.16–15.15)
**Recovery ≥1000**	Default (time range midpoints)	Indirect action	1.78 (0.52–6.32)
**Recovery ≥1000**	Earliest possible action, latest possible threshold crossing	Indirect action	1.77 (0.51–6.52)
**Recovery ≥1000**	Latest possible action, earliest possible threshold crossing	Indirect action	1.77 (0.52–6.37)
**Recovery ≥1000**	Default (time range midpoints)	Limiting factor mitigation	3.81 (1.01–15.86)
**Recovery ≥1000**	Earliest possible action, latest possible threshold crossing	Limiting factor mitigation	3.76 (1.02–14.08)
**Recovery ≥1000**	Latest possible action, earliest possible threshold crossing	Limiting factor mitigation	3.79 (0.99–14.95)
**Recovery ≥1000**	Default (time range midpoints)	Population manipulation	3.23 (0.87–12.92)
**Recovery ≥1000**	Earliest possible action, latest possible threshold crossing	Population manipulation	3.23 (0.92–12.46)
**Recovery ≥1000**	Latest possible action, earliest possible threshold crossing	Population manipulation	3.28 (0.88–12.64)
**Recovery ≥250**	Default (time range midpoints)	Indirect action	1.22 (0.42–3.48)
**Recovery ≥250**	Earliest possible action, latest possible threshold crossing	Indirect action	1.23 (0.42–3.48)
**Recovery ≥250**	Latest possible action, earliest possible threshold crossing	Indirect action	1.23 (0.42–3.62)
**Recovery ≥250**	Default (time range midpoints)	Limiting factor mitigation	7.56 (2.51–24.83)
**Recovery ≥250**	Earliest possible action, latest possible threshold crossing	Limiting factor mitigation	7.64 (2.49–24.12)
**Recovery ≥250**	Latest possible action, earliest possible threshold crossing	Limiting factor mitigation	7.96 (2.62–25.86)

**Recovery ≥250**	Default (time range midpoints)	Population manipulation	5.94 (2.03–18.41)
**Recovery ≥250**	Earliest possible action, latest possible threshold crossing	Population manipulation	5.90 (1.98–17.75)
**Recovery ≥250**	Latest possible action, earliest possible threshold crossing	Population manipulation	5.27 (1.82–15.19)

S4. Association between IUCN threat category and action and recovery status

Table S4a IUCN threat category cross-tabulation

Threat category	Species (n)	Percent of total	Percent indirect	Percent limiting factor mitigation	Percent population manipulation	Percent any direct action	Percent extinct	Percent recovered ≥100
**Residential & commercial development**	7	5	57.1	14.3	14.3	14.3	57.1	0
**Geological events**	1	0.7	0	0	0	0	100	0
**Climate change & severe weather**	9	6.4	77.8	33.3	22.2	33.3	44.4	22.2
**Agriculture & aquaculture**	36	25.5	63.9	22.2	22.2	25	36.1	11.1
**Energy production & mining**	2	1.4	50	50	100	100	0	0
**Transportation & service corridors**	4	2.8	100	50	50	50	0	25
**Biological resource use**	43	30.5	58.1	27.9	27.9	32.6	27.9	23.3
**Human intrusions & disturbance**	5	3.5	60	40	40	40	20	20
**Natural system modifications**	14	9.9	57.1	21.4	21.4	28.6	42.9	14.3

**Invasive & problematic species**	46	32.6	37	30.4	41.3	47.8	47.8	21.7
**Pollution**	4	2.8	25	0	0	0	75	0

Table S4b IUCN threat enrichment test

For each threat category causing ongoing or historical decline of at least "Slow, Significant Declines" severity over the majority of the population of 10 or more species, we tested association with direct action and with recovery via Fisher's exact test on species-level 2×2 tables, with Benjamini-Hochberg correction within each outcome. No category showed a significant association with either outcome after correction (all $q \geq 0.23$).

Category	Outcome	Species (n)	p	q	OR
**Invasive & problematic species**	Any direct action	46	0.06	0.23	2.07 (0.95-4.58)
**Agriculture & aquaculture**	Any direct action	36	0.11	0.23	0.5 (0.19-1.24)
**Biological resource use**	Any direct action	43	0.58	0.77	0.8 (0.34-1.8)
**Natural system modifications**	Any direct action	14	0.77	0.77	0.68 (0.15-2.54)
**Agriculture & aquaculture**	Recovered	36	0.07	0.26	0.33 (0.08-1.05)
**Natural system modifications**	Recovered	14	0.52	1	0.52 (0.05-2.53)
**Invasive & problematic species**	Recovered	46	0.83	1	0.87 (0.33-2.15)
**Biological resource use**	Recovered	43	1	1	0.99 (0.38-2.46)

References

1. IUCN. 2025 The IUCN Red List of Threatened Species. *IUCN Red List of Threatened Species. Version 2025-2*. See https://www.iucnredlist.org/en (accessed on 3 February 2026).

2. Pickrell J. 2018 Rare Tree Kangaroo Reappears After Vanishing for 90 Years. *National Geographic*. See https://www.nationalgeographic.com/animals/2018/09/rare-wondiwoi-tree-kangaroo-discovered-mammals-animals/ (accessed on 29 January 2021).

3. Andrade-Sánchez J, Mellink E, Riojas-López ME, Tremor S, Vanderplank SE. 2023 Site occupation and range expansion by the endangered, Mexican microendemic San Quintín Kangaroo Rat (Dipodomys gravipes). *J Mammal* **105**, 168–174. (doi:10.1093/jmammal/gyad113)

4. Rayner MJ, Gaskin CP, Taylor GA, Tennyson AJD, Fitzgerald NB, Baird KA, Friesen MR, Ross J, Ismar-Rebitz SMH. 2020 Population estimation of the New Zealand storm petrel (Fregetta maoriana) from mark-recapture techniques at Hauturu/Little Barrier Island and from at-sea resightings of banded birds. *Notornis* **67**, 503. (doi:10.63172/062223dqwebs)

5. Bailey CA, Sefczek TM, Robertson BAD, Rasoamazava L, Rakotomalala VF, Andriamadison JDN, Randrianasolo F, Andriajaona A, Louis EE. 2020 A Re-evaluation of the Northern Sportive Lemur (Lepilemur septentrionalis) Population at Montagne des Français, and a Review of Its Current State of Conservation in the Protected Area. *Primate Conservation* **34**, 53–59.

6. Ryan PG, Moloney CL, Dilley BJ, Glass T, Schofield A. 2024 The impact of storm-induced tree loss on the population of Wilkins's Finch Nesospiza wilkinsi. *Bird Conservation International* **34**, e38. (doi:10.1017/S0959270924000303)

7. Lu Y *et al.* 2020 Creating a conservation network: Restoration of the critically endangered Chinese crested tern using social attraction. *Biological Conservation* **248**, 108694. (doi:10.1016/j.biocon.2020.108694)

8. David SB, Dolev A, Parchizadeh J, Avni-Magen N, Saltz D. 2025 Persian Fallow Deer Dama mesopotamica (Brooke, 1875). In *Deer of the World: Ecology, Conservation and Management* (eds M Melletti, S Focardi), pp. 109–120. Cham: Springer Nature Switzerland. (doi:10.1007/978-3-031-17756-9_5)

9. Smith D *et al.* 2023 Extinct in the wild: The precarious state of Earth's most threatened group of species. *Science* **379**, eadd2889. (doi:10.1126/science.add2889)

10. Pucek Z, editor. 2004 *European bison: status survey and conservation action plan*. Gland, Switzerland ; Cambridge: IUCN/SSC Bison Specialist Group.

11. USFWS. 2019 Species Status Assessment Report for the Black-footed Ferret (Mustela nigripes).

12. USFWS. 2013 Recovery plan for the black-footed ferret (Mustela nigripes). , 157 pp.

13. Spalton A. 1993 A brief history of the reintroduction of the Arabian oryx Oryx leucoryx into Oman 1980–1992. *International Zoo Yearbook* **32**, 81–90. (doi:10.1111/j.1748-1090.1993.tb03519.x)

14. Cheng Z *et al.* 2021 Reintroduction, distribution, population dynamics and conservation of a species formerly extinct in the wild: A review of thirty-five years of successful Milu (Elaphurus davidianus) reintroduction in China. *Global Ecology and Conservation* **31**, e01860. (doi:10.1016/j.gecco.2021.e01860)

15. Turvey ST, Barnes I, Marr M, Brace S. 2017 Imperial trophy or island relict? A new extinction paradigm for Père David's deer: a Chinese conservation icon. *Royal Society Open Science* **4**, 171096. (doi:10.1098/rsos.171096)

16. USFWS. 2025 California Condor Recovery Program 2024 Annual Population Status.

17. USFWS. 1996 California Condor Recovery Plan, Third Revision.

18. Wakefield S, Knowles J, Zimmermann W, van Dierendonck M. 2002 Status and Action Plan for the Przewalski's Horse (Equus ferus przewalskii). In *Equids : zebras, asses and horses : status survey and conservation action plan* (ed PD Moehlman), pp. 82–92. Gland, Switzerland: IUCN.

19. Whitemore S, Gilbert T. 2024 International studbook for the scimitar-horned oryx Oryx dammah.

20. Jones CG. 1987 The larger land-birds of Mauritius. In *Studies of Mascarene Island Birds* (ed AW Diamond), pp. 208–300. Cambridge: Cambridge University Press. (doi:10.1017/CBO9780511735769.007)

21. Ma L *et al.* 2021 Changes in the Habitat Preference of Crested Ibis (Nipponia nippon) during a Period of Rapid Population Increase. *Animals* **11**. (doi:10.3390/ani11092626)

22. Sun Y, Skidmore AK, Wang T, Gils HAMJ van, Wang Q, Qing B, Ding C. 2014 Reduced Dependence of Crested Ibis on Winter-Flooded Rice Fields: Implications for Their Conservation. *PLOS ONE* **9**, e98690. (doi:10.1371/journal.pone.0098690)

23. Ardern SL, Lambert DM. 1997 Is the black robin in genetic peril? *Molecular Ecology* **6**, 21–28. (doi:10.1046/j.1365-294X.1997.00147.x)

24. Swinnerton KJ, Groombridge JJ, Jones CG, Burn RW, Mungroo Y. 2004 Inbreeding depression and founder diversity among captive and free-living populations of the endangered pink pigeon Columba mayeri. *Animal Conservation* **7**, 353–364. (doi:10.1017/S1367943004001556)

25. Cheke A. 1979 The Rodrigues Fody Foudia flavicans. A brief history of its decline, and a report on the 1978 expedition. *Dodo (Rep. Jersey Wildl. Pres. Trust)* **15**, 12–19.

26. Cavill EL *et al*. 2022 Conservation genomics of the endangered Seychelles Magpie-Robin (Copsychus sechellarum): a unique insight into the history of a precious endemic bird. *Ibis* **164**, 396–410. (doi:10.1111/ibi.13023)

27. Watson J, Warman C, Todd D, Laboudallon V. 1992 The Seychelles magpie robin Copsychus sechellarum: ecology and conservation of an endangered species. *Biological Conservation* **61**, 93–106. (doi:10.1016/0006-3207(92)91099-E)

28. USFWS. 2023 Puerto Rican Parrot (Amazona vittata) 5-Year Review: Summary and Evaluation.

29. NESPTSRH. 2018 Threatened Species Strategy Year 3 Scorecard – Gilbert's Potoroo.

30. DPW. 2016 Gilbert's potoroo (Potorous gilbertii) Recovery Plan.

31. MWF. 1997 Echo Parakeet (Psittacula eques) Management Report 1997.

32. McAbee K, Conkin J. 2025 Whooping Crane Status. 2023 Breeding Season to 2024 Spring Migration.

33. CWS, USFWS. 2007 International recovery plan for the whooping crane.

34. 2025 Householder - Woodhen survey results. *Lord Howe Island Board*. See https://www.lhib.nsw.gov.au/news/householder-woodhen-survey-results (accessed on 19 December 2025).

35. Portelli D, Carlile N. 2019 Preventing the extinction of the Lord Howe Woodhen (Hypotaenidia sylvestris) through predator eradication and population augmentation. *Ornithological Science* **18**, 169–175. (doi:10.2326/osj.18.169)

36. USFWS. 2008 Short-tailed Albatross Recovery Plan.

37. Reed C, Murray DP. 1993 Black Stilt Recovery Plan (Himantopus novaezealandiae).

38. Te Ipukarea Society. 2020 Kakerori Species Status Report 2020.

39. Robertson HA, Saul EK. 2007 Conservation of kakerori (Pomarea dimidiata) in the Cook Islands in 2005/06.

40. Environment and Climate Change Canada. 2020 Recovery Strategy for the Vancouver Island Marmot (Marmota vancouverensis) in Canada.

41. 2015 Vancouver Island Marmot Population and Habitat Viability Assessment Workshop Final Report.

42. Showler DA, Côté IM, Jones CG. 2002 Population census and habitat use of Rodrigues Warbler Acrocephalus rodericanus. *Bird Conservation International* **12**, 211–230. (doi:10.1017/S0959270902002137)

43. Komdeur J. 1994 Conserving the seychelles warbler Acrocephalus sechellensis by translocation from Cousin Island to the islands of Aride and Cousine. *Biological Conservation* **67**, 143–152. (doi:10.1016/0006-3207(94)90360-3)

44. Groombridge JJ, Dawson DA, Burke T, Prys-Jones R, Brooke M de L, Shah N. 2009 Evaluating the demographic history of the Seychelles kestrel (*Falco araea*): Genetic evidence for recovery from a population bottleneck following minimal conservation management. *Biological Conservation* **142**, 2250–2257. (doi:10.1016/j.biocon.2009.04.026)

45. Smith JD. 1952 The Hawaiian Goose (Nene) Restoration Program. *The Journal of Wildlife Management* **16**, 1–9. (doi:10.2307/3797173)

46. USFWS. 2004 Draft Revised Recovery Plan for the Nēnē or Hawaiian Goose (Branta sandvicensis).

47. Madeiros J. 2025 2024/25 Cahow Season Wrap Up. *The Nonsuch Expeditions*. See https://www.nonsuchisland.com/blog/202425-cahow-season-wrap-up (accessed on 14 December 2025).

48. Madeiros J. 2005 Recovery Plan For The Bermuda Petrel (Cahow) Pterodroma cahow.

49. SOP Manu. 2025 Activity Report 2024.

50. Blanvillain C, Ghestemme T, Saavedra S, Yan L, Michoud-Schmidt J, Beaune D, O'Brien M. 2020 Rat and invasive birds control to save the Tahiti monarch (*Pomarea nigra*), a critically endangered island bird. *Journal for Nature Conservation* **55**, 125820. (doi:10.1016/j.jnc.2020.125820)

51. Hooson S, Jamieson IG. 2003 The distribution and current status of New Zealand Saddleback Philesturnus carunculatus. *Bird Conservation International* **13**, 79–95. (doi:10.1017/S0959270903003083)

52. DES. 2022 Northern Hairy-Nosed Wombat Recovery Action Plan 2022.

53. Crossman DG, Johnsow CN, Horsup AB. 1994 Trends in the population of the Northern Hairy-nosed Wombat Lasiorhinus krefftii in Epping Forest National Park, Central Queensland. *Pac Conserv Biol* **1**, 141–149. (doi:10.1071/PC940141)

54. Hartmann SA, Segelbacher G, Juiña ME, Schaefer HM. 2015 Effects of habitat management can vary over time during the recovery of an endangered bird species. *Biological Conservation* **192**, 154–160. (doi:10.1016/j.biocon.2015.09.014)

55. Gummer H, Williams M. 1999 Campbell Island Teal: conservation update. *Wildfowl* **50**, 133–138.
